# Correction: Belchior et al. Repair Kinetics of DSB-Foci Induced by Proton and α-Particle Microbeams of Different Energies. *Life* 2022, *12*, 2040

**DOI:** 10.3390/life14010036

**Published:** 2023-12-25

**Authors:** Ana Belchior, João F. Canhoto, Ulrich Giesen, Frank Langner, Hans Rabus, Reinhard Schulte

**Affiliations:** 1 Centro de Ciências e Tecnologias Nucleares, Instituto Superior Técnico, Universidade de Lisboa, Estrada Nacional 10 (km 139,7), 2695-066 Bobadela LRS, Portugal; joaofcanhoto@tecnico.ulisboa.pt; 2 Departamento de Física, Instituto Superior Técnico, Universidade de Lisboa, Av. Rovisco Pais, 1049-001 Lisboa, Portugal; 3 Physikalisch-Technische Bundesanstalt (PTB), 38116 Braunschweig, Germany; ulrich.giesen@ptb.de (U.G.); frank.langner@ptb.de (F.L.); 4 Physikalisch-Technische Bundesanstalt (PTB), 10587 Berlin, Germany; hans.rabus@ptb.de; 5 Division of Biomedical Engineering Sciences, Loma Linda University, Loma Linda, CA 92350, USA; rschulte@llu.edu

## Error in Table

In the original publication [1], there was a mistake in Table 3 as published. In one of the cells of this table (column 5, row labelled “α—particles 20 MeV”), the same values as in the cell below (column 5, row labelled “α—particles 10 MeV”) were shown. The corrected Table 3 appears below.

The authors state that the scientific conclusions are unaffected. This correction was approved by the Academic Editor. The original publication has also been updated.

## Figures and Tables

**Table 3:** Results of the model parameters obtained via simultaneous non-linear regression of all datasets: number of radiation-induced foci per track ${\bar{n}}_{Q}$ , fraction of persistent radiation-induced foci $p_{Q}$ , mean number of persistent radiation-induced foci per track ${\bar{p}}_{Q},$ repair rates $\beta_{1}$ and $\beta_{2}$ , and respective standard errors (SEs) obtained from the fit of the non-linear model (Equations (1) to (3)) to the ensemble of datasets for all radiation qualities and the sham-irradiated cells. The values are from the regression performed using the GDL MPfit procedure imposing $\beta_{0}=\beta_{1}$ . The upper and lower values in the cells in columns 3 through 7 are the fit results obtained using Equations (4) and (5), respectively, in conjunction with Equations (1) to (3). The values given in italics in columns 4 and 5 were calculated from the values in the respective other column. The resulting ratio $\chi^{2}/f$ of the weighted sum of squared residuals $\chi^{2}$ (summed over all datasets) to the degrees of freedom f is about 4.8 in both cases. In columns 6 and 7, only one value is given since, in the simultaneous fit, these parameters were kept the same for all radiation qualities.

(1) Radiation Beam	(2) LET (keV/µm)	(3) Mean Number of Foci Per Track, ${\bar{n}}_{Q\;}$	(4) Proportion of Persistent Foci, $p_{Q}$	(5) Mean Number of Persistent Foci Per Track, ${\bar{p}}_{Q\;}\;$	(6) Repair Rate $\beta_{1}\;\left(h^{-1}\right)$	(7) Repair Rate $\beta_{2}\;\left(h^{-1}\right)$
Protons 3 MeV	19 ± 2	0.37 ± 0.02 0.37 ± 0.02	0.42 ± 0.06 *0.38 ± 0.05*	*0.15 ± 0.02* 0.14 ± 0.02	0.43 ± 0.01 0.41 ± 0.01	0.06 ± 0.01 0.05 ± 0.01
α—particles 20 MeV	36 ± 1	0.69 ± 0.04 0.69 ± 0.04	0.25 ± 0.06 *0.22 ± 0.05*	*0.17 ± 0.04* 0.15 ± 0.03		
10 MeV	85 ± 4	1.13 ± 0.06 1.13 ± 0.06	0.33 ± 0.06 *0.30 ± 0.05*	*0.38 ± 0.08* 0.34 ± 0.06		
8 MeV	170 ± 40	1.68 ± 0.18 1.68 ± 0.18	0.31 ± 0.08 *0.27 ± 0.07*	*0.52 ± 0.15* 0.47 ± 0.10		
