# Supplementary material for: Repair Kinetics of DSB-Foci Induced by Proton and α-Particle Microbeams of Different Energies
Source: Life (Basel). 2022 Dec 6;12(12):2040. doi: 10.3390/life12122040 (PMC9785158; doi:10.3390/life12122040)
Supplement: Supplementary file 1 [file life-12-02040-s001.zip › life-2028553-supplementary.pdf]

Supplementary Information

Supplementary Table S1. Summary of the approach used in the simulation of ion track clustering that results in indistinguishable foci: The scatter of the actual ion position around the target position was sampled from a two-dimensional Gaussian distribution. The parameters of this distribution have been established experimentally and are given in Supplementary Table S2. Similar to [15], two ion tracks were considered as part of a cluster when their distance was below a set value (2 μm). Different from the approach of [15], the occurrence of zero or two ions emitted when the fluorescence detection system at the microbeam registers the passage of an ion has been included in this simulation and the ion pairs were further analyzed to identify clusters of more than two ion tracks. The cell nuclei were assumed to be of elliptical shape. In contrast to [15], it was taken into account that the half-axes found in the measured nuclei are correlated. Therefore, a principal components analysis (PCA) of the bi-variate distribution of long and short half axes was performed to obtain independently distributed variables that were found to be well approximated by Gaussian distributions. Therefore, the principal components c_1 and c_2 were randomly sampled from these Gaussian distributions and the long and short half axes were obtained as $a = \bar{a} + v_1 c_1 - v_2 c_2$ and $b = \bar{b} + v_2 c_1 + v_1 c_2$, where (v_1, v_2) is the first eigenvector of the PCA and $\bar{a}$ and $\bar{b}$ are the mean values of the distributions of long and short half axes observed with the scored cell nuclei.

Input quantity	Sampling distribution
cell nucleus properties	
equivalent ellipse half axes a and b	calculated from sampled principal components
principal components of bi-variate distribution of a and b	Gaussian
equivalent ellipse orientation (azimuth angle of long axis)	uniform between 0 and π
ion microbeam properties	
deviation from target position	two-dimensional Gaussian
ion passage detection	fixed values

Supplementary Table S2. Parameters used in the simulation of ion track clustering that results in indistinguishable foci. Similar to [15], two ion tracks were considered as part of a cluster when their distance was below a set value.

Radiation beam	α-particles 20 MeV	α-particles 10 MeV	α-particles 8 MeV
beam			
horizontal FWHM / μm	4.2	4.5	4.5
vertical FWHM / μm	3.9	3.5	3.5
noise event frequency [a]	1%	1%	0.1%
double event frequency [b]	1%	1%	1%
cell nuclei			
mean long half axis / μm	8.31	8.28	7.73
mean short half axis / μm	5.54	5.54	5.53

sigma principal component 1 / μm	1.20	1.27	1.23
sigma principal component 2 / μm	0.53	0.55	0.55
first eigenvector	(0.923,0.385)	(0.909,0.417)	(0.897,0.442)

[a] Noise event: the fluorescence detection system at the microbeam records the passage of an ion while no ion is emitted

[b] Double event: An ion passes the exit window of the microbeam producing a fluorescence signal below threshold, such that when the fluorescence detection system records the passage of an ion, in reality two ions have been emitted

Supplementary Table S3. Values used in eq. (3) for the mean number of clusters of tracks that result in indistinguishable foci. For the three α-particle beams the values were obtained by simulations using 10^6 random samples of the positions of the ion trajectories and of nuclei dimensions and orientations. The values for protons are the averages of the three values for the α-particles. Similar to [15], two foci were assumed to be indistinguishable when their distance was below 2 μm.

Radiation beam	protons 3 MeV	α-particles 20 MeV	α-particles 10 MeV	α-particles 8 MeV
isolated tracks	3.0371	3.0435	3.0312	3.0366
cluster of 2 tracks	0.5916	0.5890	0.5910	0.5949
cluster of 3 tracks	0.1500	0.1491	0.1496	0.1515
cluster of 4 tracks	0.0305	0.0305	0.0302	0.0307
cluster of 5 tracks	0.0044	0.0046	0.0041	0.0045
cluster of 6 tracks	0.0001	0.0001	0.0002	0.0001

Supplementary Table S4. Results for the uncertainty of the mean number of clusters of tracks that result in indistinguishable foci. The values were obtained from simulations performed for the three α-particle beams with 1000 batches of 1000 random samples of the positions of the ion trajectories and of nuclei dimensions and orientations. The values listed are the mean and the sample standard deviation of the average values per batch. Similar to [15], two foci were assumed to be indistinguishable when their distance was below 2 μm.

Radiation beam	α-particles 20 MeV	α-particles 10 MeV	α-particles 8 MeV
isolated tracks	3.042 ± 0.029	3.039 ± 0.044	3.033 ± 0.044
cluster of 2 tracks	0.586 ± 0.021	0.588 ± 0.020	0.594 ± 0.021
cluster of 3 tracks	0.150 ± 0.012	0.149 ± 0.011	0.152 ± 0.012
cluster of 4 tracks	0.0312 ± 0.0057	0.0306 ± 0.054	0.0313 ± 0.0054
cluster of 5 tracks	0.0044 ± 0.0021	0.00042 ± 0.00021	0.0044 ± 0.0002
cluster of 6 tracks	0.00018 ± 0.00043	0.00015 ± 0.00039	0.00018 ± 0.00043

Supplementary Table S5. Results for the mean and standard deviation of the number of clusters of tracks that results in indistinguishable foci for α-particles of 20 MeV beam energy. Similar to [15], two foci were assumed to be indistinguishable when their distance was below 2 μm. The values in the second column are the mean and the sample standard deviation among the means

of the free model parameters obtained by non-linear regression using values for the parameters $\bar{k}_Q$ determined from 1000 batches containing each 1000 samples of the positions of the ion trajectories and of nuclei dimensions and orientations. The last column shows the fit results and estimated uncertainties of the free model parameters from the last batch. The uncertainties given in this column are representative for the uncertainties due to the non-linear regression for a particular set of parameters $\bar{k}_Q$. The uncertainties from the scatter of the parameters $\bar{k}_Q$ are negligible compared to the uncertainties from the fit procedure.

parameter	all batches	last batch
$\bar{n}_{Q,1}$	0.403 ± 0.003	0.404 ± 0.044
$\bar{n}_{Q,2}$	0.647 ± 0.005	0.645 ± 0.089
$\bar{n}_{Q,3}$	1.124 ± 0.012	1.11 ± 0.14
$\bar{n}_{Q,4}$	1.769 ± 0.027	1.80 ± 0.44
$p_{Q,1}$	0.1336 ± 0.0009	0.13 ± 0.25
$p_{Q,2}$	0.0724 ± 0.0006	0.07 ± 0.14
$p_{Q,3}$	0.0789 ± 0.0013	0.08 ± 0.15
$p_{Q,4}$	0.0811 ± 0.0014	0.08 ± 0.16
n_b	0.1459 ± 0.0001	0.15 ± 0.09
n_s	0.5464 ± 0.0004	0.55 ± 0.15
β_0 (h^{-1})	0.4372 ± 0.0002	0.437 ± 0.015
β_1 (h^{-1})	0.2500 ± 0.0005	0.25 ± 0.10
β_2 (h^{-1})	0.0001 ± 0.0003	0.000 ± 0.085

Supplementary Table S6. Results of the model parameters obtained by separate non-linear regression of each dataset: Mean number of radiation-induced foci per track $\bar{n}_Q$, fraction of persistent radiation-induced foci p_Q, mean number of persistent radiation-induced foci per track $\bar{p}_Q$ (calculated from $\bar{n}_Q$ and p_Q), repair rates β_1 and β_2, and respective standard errors (SE) obtained from the fit of the non-linear model (equation 3) to the difference of the data of a radiation quality and the sham-irradiated cells. The values are from the regression performed using the MPfit procedure of GDL.

Radiation beam	LET (keV/μm)	Mean number of foci per track, $\bar{n}_Q$	Proportion of persistent radiation-induced foci, p_Q	Mean number of persistent foci per track, $\bar{p}_Q$	Repair rate, β_1 (h^{-1})	Repair rate, β_2 (h^{-1})
protons						
3 MeV	19 ± 2	0.37 ± 0.04	0.11 ± 0.06	0.04 ± 0.02	0.34 ± 0.11	0.00 ± 0.00
α – particles						
20 MeV	36 ± 1	0.89 ± 0.16	0.38 ± 0.15	0.34 ± 0.15	0.96 ± 0.56	0.09 ± 0.04
10 MeV	85 ± 4	1.53 ± 0.45	0.43 ± 0.19	0.66 ± 0.34	1.11 ± 0.92	0.09 ± 0.03
8 MeV	170 ± 40	1.70 ± 0.47	0.27 ± 0.27	0.47 ± 0.48	0.43 ± 0.44	0.05 ± 0.06

Supplementary Table S7. Results of the model parameters obtained by separate non-linear regression of each dataset: Mean number of radiation-induced foci per track $\bar{n}_Q$, fraction of persistent radiation-induced foci p_Q, mean number of persistent radiation-induced foci per track $\bar{p}_Q$ (calculated from $\bar{n}_Q$ and p_Q), repair rate β_1, and respective standard errors (SE) obtained from the fit of the non-linear model (equation 3) to the difference of the data of a radiation quality and the sham-irradiated cells. The values are from the regression performed using the MPfit procedure of GDL imposing $\beta_2 = 0$.

Radiation beam	LET (keV/μm)	Mean number of foci per track, $\bar{n}_Q$	Proportion of persistent foci, p_Q	Mean number of persistent foci	Repair rate, β_1 (h^{-1})
protons					
3 MeV	19 ± 2	0.37 ± 0.04	0.11 ± 0.06	0.04 ± 0.02	0.34 ± 0.11
α – particles					
20 MeV	36 ± 1	0.73 ± 0.10	0.07 ± 0.04	0.07 ± 0.03	0.26 ± 0.06
10 MeV	85 ± 4	1.17 ± 0.21	0.06 ± 0.04	0.07 ± 0.05	0.21 ± 0.06
8 MeV	170 ± 40	1.62 ± 0.36	0.09 ± 0.05	0.15 ± 0.09	0.26 ± 0.08

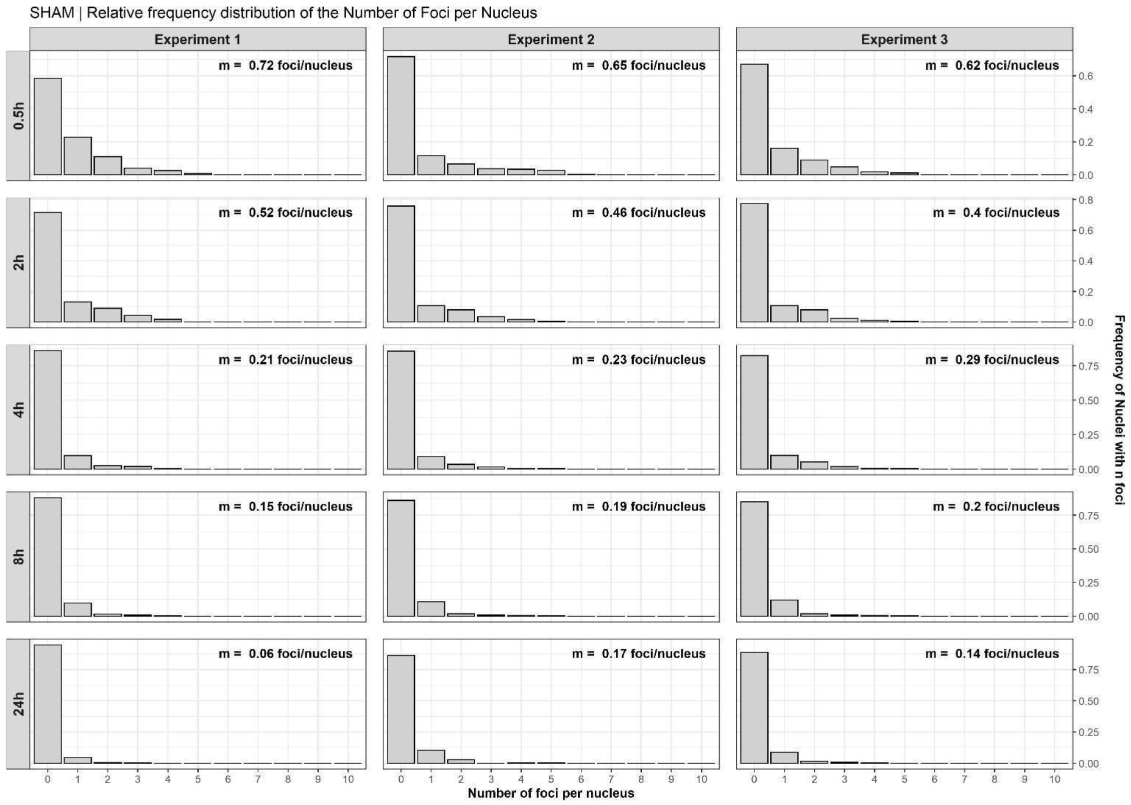

Supplementary Figure S1. Relative frequency distribution of the number of 53BP1 foci per nucleus for sham-treated cells, for three replicate experiments and for each timepoint after scanning. Inside each histogram, the mean number of foci per nucleus, m, is indicated.

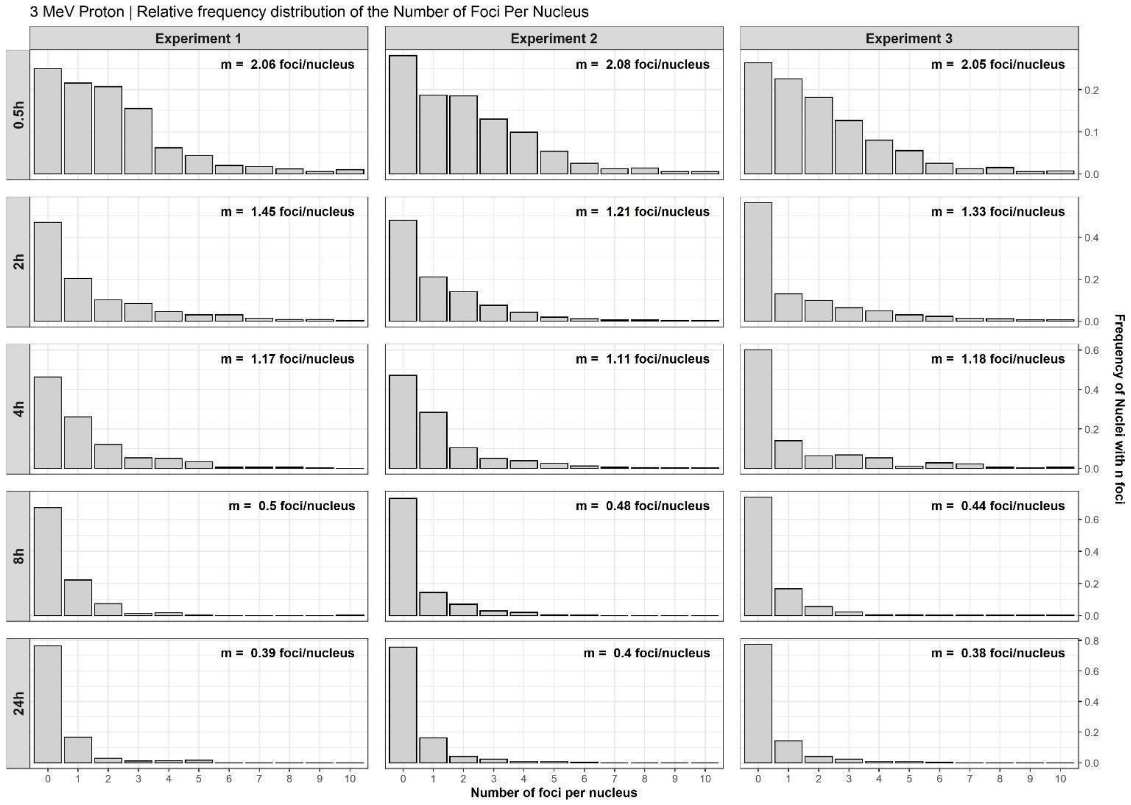

Supplementary Figure S2. Relative frequency distribution of the number of 53BP1 foci per nucleus for cells irradiated with 3 MeV Protons, for three replicate experiments and for each timepoint after irradiation. Inside each histogram, the mean number of foci per nucleus, m, is indicated.

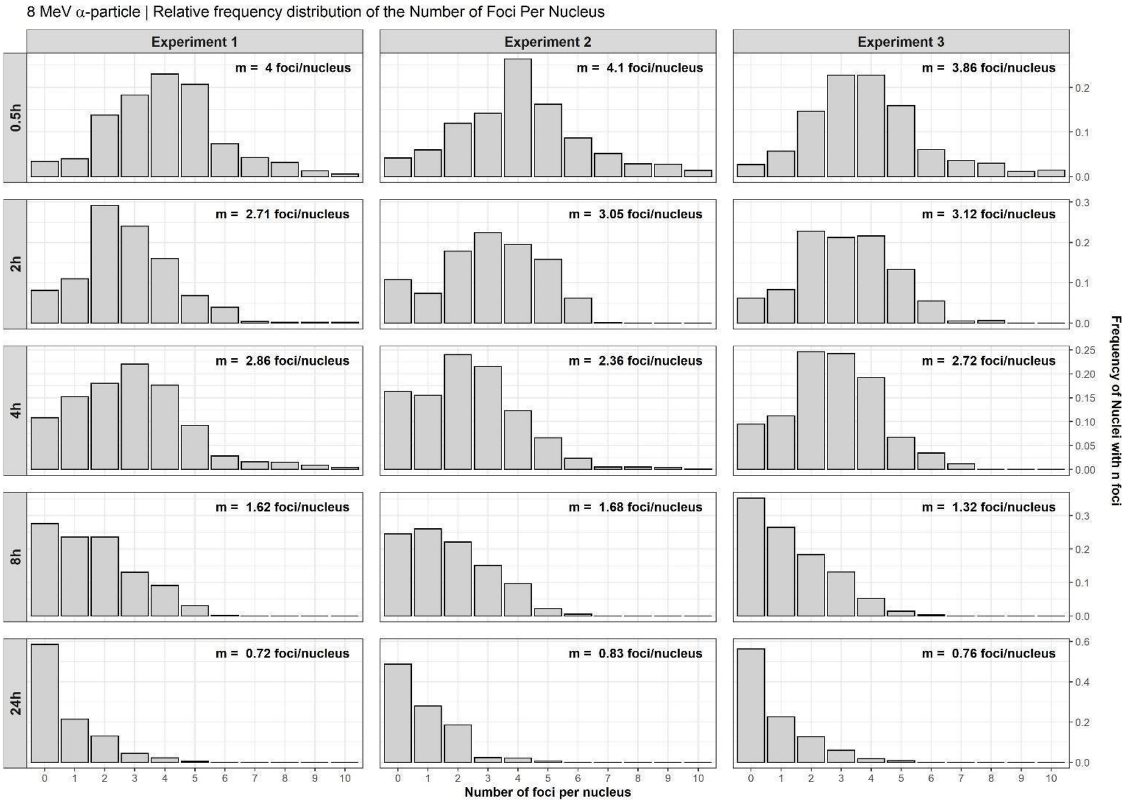

Supplementary Figure S3. Relative frequency distribution of the number of 53BP1 foci per nucleus for cells irradiated with 8 MeV α-particles, for three replicate experiments and for each timepoint after irradiation. Inside each histogram, the mean number of foci per nucleus, m, is indicated.

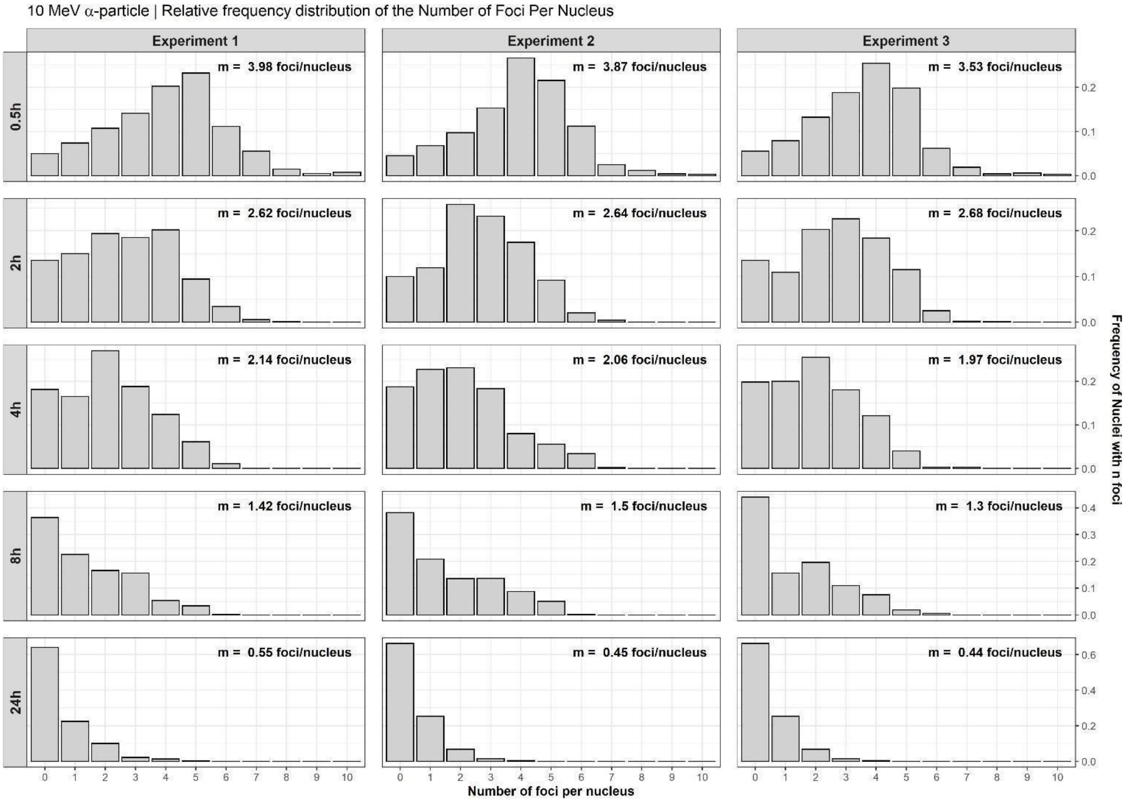

Supplementary Figure S4. Relative frequency distribution of the number of 53BP1 foci per nucleus for cells irradiated with 10 MeV α-particles, for three replicate experiments and for each timepoint after irradiation. Inside each histogram, the mean number of foci per nucleus, m, is indicated.

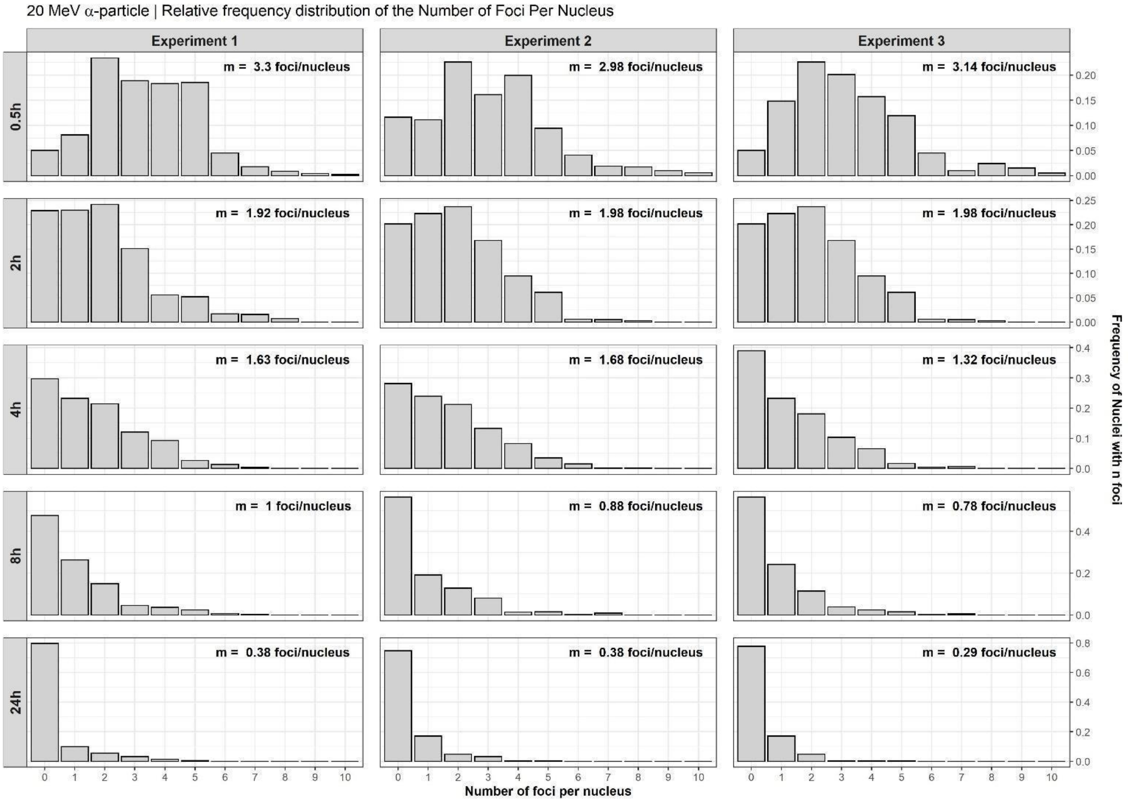

Supplementary Figure S5. Relative frequency distribution of the number of 53BP1 foci per nucleus for cells irradiated with 20 MeV α-particles, for three replicate experiments and for each timepoint after irradiation. Inside each histogram, the mean number of foci per nucleus, m, is indicated.
